# Agronomic nitrogen‐use efficiency of rice can be increased by driving *OsNRT2.1* expression with the *OsNAR2.1* promoter

**DOI:** 10.1111/pbi.12531

**Published:** 2016-01-30

**Authors:** Jingguang Chen, Yong Zhang, Yawen Tan, Min Zhang, Longlong Zhu, Guohua Xu, Xiaorong Fan

**Affiliations:** ^1^ State Key Laboratory of Crop Genetics and Germplasm EnhancementMinistry of AgricultureNanjing Agricultural UniversityNanjingChina; ^2^ Key Laboratory of Plant Nutrition and Fertilization in Low‐Middle Reaches of the Yangtze RiverMinistry of AgricultureNanjing Agricultural UniversityNanjingChina

**Keywords:** *OsNAR2.1* promoter, *Oryza sativa*, *OsNRT2.1*, agronomic nitrogen‐use efficiency

## Abstract

The importance of the nitrate (${\text{NO}}_{3}^{-}$) transporter for yield and nitrogen‐use efficiency (NUE) in rice was previously demonstrated using map‐based cloning. In this study, we enhanced the expression of the *OsNRT2.1* gene, which encodes a high‐affinity ${\text{NO}}_{3}^{-}$ transporter, using a ubiquitin (*Ubi*) promoter and the ${\text{NO}}_{3}^{-}$‐inducible promoter of the *OsNAR2.1* gene to drive *OsNRT2.1* expression in transgenic rice plants. Transgenic lines expressing *pUbi:OsNRT2.1* or *pOsNAR2.1:OsNRT2.1* constructs exhibited the increased total biomass including yields of approximately 21% and 38% compared with wild‐type (WT) plants. The agricultural NUE (ANUE) of the *pUbi:OsNRT2.1* lines decreased to 83% of that of the WT plants, while the ANUE of the *pOsNAR2.1:OsNRT2.1* lines increased to 128% of that of the WT plants. The dry matter transfer into grain decreased by 68% in the *pUbi:OsNRT2.1* lines and increased by 46% in the *pOsNAR2.1:OsNRT2.1* lines relative to the WT. The expression of *OsNRT2.1* in shoot and grain showed that *Ubi* enhanced *OsNRT2.1* expression by 7.5‐fold averagely and *OsNAR2.1* promoters increased by about 80% higher than the WT. Interestingly, we found that the *OsNAR2.1* was expressed higher in all the organs of *pUbi:OsNRT2.1* lines; however, for *pOsNAR2.1:OsNRT2.1* lines, *OsNAR2.1* expression was only increased in root, leaf sheaths and internodes. We show that increased expression of *OsNRT2.1*, especially driven by *OsNAR2.1* promoter, can improve the yield and NUE in rice.

## Introduction

Rice (*Oryza sativa* L.) is not only a major staple food crop for a large part of the world population but also an important model monocot plant species for research because of its small genome size and the availability of the complete rice genome sequence (Feng *et al*., 2002; Sasaki *et al*., 2002). Nitrogen (N) nutrition affects all levels of plant function from metabolism to resource allocation, growth and development (Crawford, 1995; Scheible *et al*., 1997, 2004; Stitt, 1999). The most abundant source for N acquisition by plant roots is nitrate (${\text{NO}}_{3}^{-}$), which is present in naturally aerobic soils due to intensive nitrification from applied organic and fertilizer N. In contrast, ammonium (${\text{NH}}_{4}^{+}$) is the main form of available N in flooded paddy soils due to the anaerobic soil conditions (Sasakawa and Yamamoto, 1978).

${\text{NO}}_{3}^{-}$ serves as a nutrient and as a signal that induces changes in the growth and gene expression (Coruzzi and Bush, 2001; Coruzzi and Zhou, 2001; Crawford and Forde, 2002; Crawford and Glass, 1998; Kirk and Kronzucker, 2005; Kronzucker *et al*., 2000; Wang *et al*., 2000; Zhang and Forde, 2000). Two different ${\text{NO}}_{3}^{-}$ uptake systems in plants, the high‐ and low‐affinity ${\text{NO}}_{3}^{-}$ uptake systems designated as HATS and LATS, respectively, are regulated by ${\text{NO}}_{3}^{-}$ supply and enable plants to cope with high or low ${\text{NO}}_{3}^{-}$ concentrations in soils (Fan *et al*., 2005).

Some high‐affinity ${\text{NO}}_{3}^{-}$ transporters belonging to the NRT2 family have been shown to require a partner protein, NAR2, for their function (Xu *et al*., 2012). Quesada *et al*. (1994) identified the *CrNar2* gene, which encodes a small protein of approximately 200 amino acid residues and which has no known transport activity, but is required for complementation of ${\text{NO}}_{3}^{-}$ transport in *Chlamydomonas reinhardtii* mutants defective in uptake. In *Arabidopsis*, Okamoto *et al*. (2006) showed that both constitutive and ${\text{NO}}_{3}^{-}$‐inducible HATS, but not LATS, depended on the expression of the NAR2‐type gene, for example *Arabidopsis AtNRT3.1*. Orsel *et al*. (2006) used yeast split‐ubiquitin and oocyte expression systems to show that *AtNAR2.1* (*AtNRT3.1*) and *AtNRT2.1* interacted to produce a functional HATS. Yong *et al*. (2010) showed that the NRT2.1 and NAR2.1 polypeptides interact directly at the plasma membrane to constitute an oligomer that may act as the functional unit for high‐affinity ${\text{NO}}_{3}^{-}$ influx in *Arabidopsis* roots. In rice, the *OsNRT2.1*,* OsNRT2.2* and *OsNRT2.3a* gene products were similarly shown to require the protein encoded by OsNAR2.1 for ${\text{NO}}_{3}^{-}$ uptake (Feng *et al*., 2011; Liu *et al*., 2014; Yan *et al*., 2011), and their interaction at the protein level was demonstrated using a yeast two‐hybrid assay and by Western blotting (Liu *et al*., 2014; Yan *et al*., 2011).

Rice seedling growth was improved slightly by increased *OsNRT2.1* expression, but N uptake remained unaffected (Katayama *et al*., 2009), probably due to the absence of the interaction with OsNAR2.1, which is required for the functional ${\text{NO}}_{3}^{-}$ transport (Feng *et al*., 2011; Yan *et al*., 2011).

In this study, we transformed the open reading frame (ORF) of the *OsNRT2.1* gene into rice with the expression driven by the *OsNAR2.1* promoter to modify the coexpression of the *OsNRT2.1* and *OsNAR2.1* genes in rice plants and to investigate the biological function of their coexpression *in vivo*. Transgenic lines expressing the *OsNRT2.1* gene under the control of the *OsNAR2.1* promoter exhibited greatly increased the growth, biomass and yield compared with transgenic lines expressing *OsNRT2.1* under a ubiquitin promoter. We analysed *OsNRT2.1* and *OsNAR2.1* expression patterns during the whole‐plant growth and show that modification of the ratio of *OsNRT2.1* to *OsNAR2.1* expression in stems altered the rice growth and agricultural N‐use efficiency (ANUE).

## Results

### Generation of transgenic rice plants expressing *pUbi:OsNRT2.1* and *pOsNAR2.1:OsNRT2.1* constructs and field analysis of traits

The ubiquitin promoter (pUbi) has been used as a strong promoter in a variety of applications in gene transfer studies and was shown to drive gene expression most actively in rapidly dividing cells (Cornejo *et al*., 1993). Overexpression of just the *OsNRT2.1* gene in rice was previously shown to not increase ${\text{NO}}_{3}^{-}$ uptake (Katayama *et al*., 2009).

We introduced *pUbi:OsNRT2.1* (Figure S1a) and *pOsNAR2.1:OsNRT2.1* (Figure S1b) expression constructs into Wuyunjing 7 (WYJ7), a rice cultivar that produces high yields in Jiangsu Province, using *Agrobacterium tumefaciens*‐mediated transformation. We generated 23 lines exhibiting increased *OsNRT2.1* expression, including 12 *pUbi:OsNRT2.1* lines and 11 *pOsNAR2.1:OsNRT2.1* lines (Figure S2).

**Figure 1:**
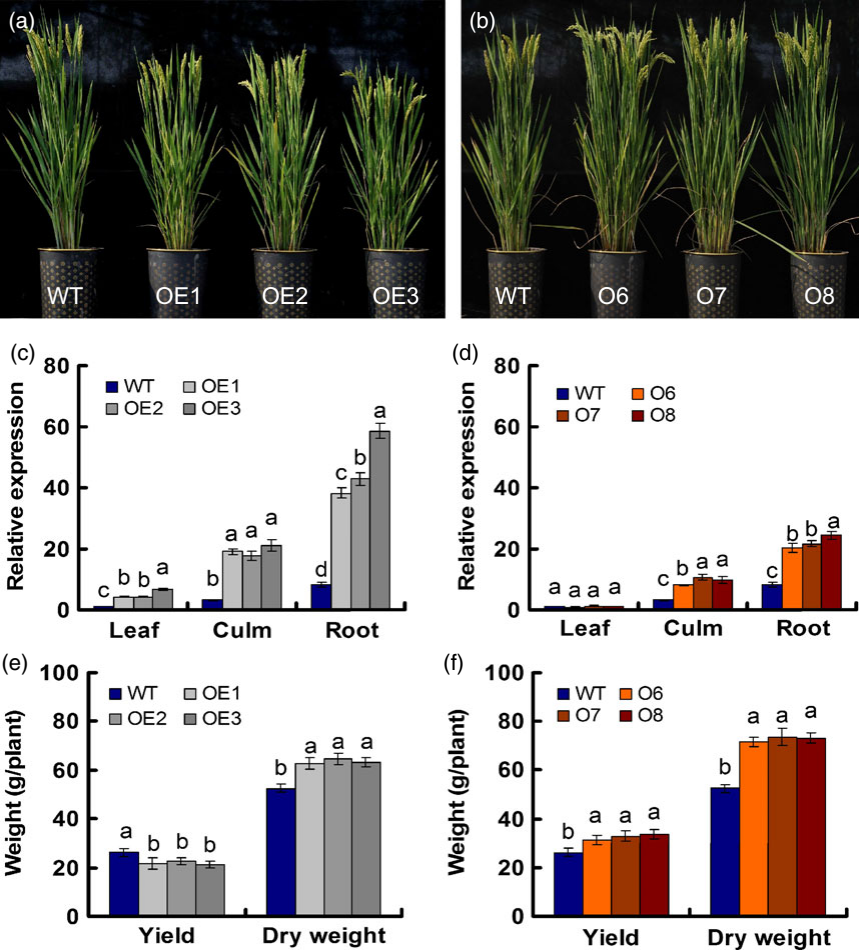
Characterization of transgenic lines. (a) Gross morphology of *pUbi:OsNRT2.1* transgenic lines (OE1, OE2 and OE3) and the wild‐type (WT). (b) Gross morphology of *pOsNAR2.1:OsNRT2.1* transgenic lines (O6, O7 and O8) and the WT. (c, d) Real‐time quantitative RT‐PCR analysis of endogenous *OsNRT2.1* expression in various transgenic lines and WT plants. (c) *pUbi:OsNRT2.1* transgenic lines (OE1, OE2 and OE3) and the WT, and (d) *pOsNAR2.1:OsNRT2.1* transgenic lines (O6, O7 and O8) and the WT. RNA was extracted from leaf blade I, culm and root. (e, f) Grain yield and dry weight per plant for transgenic and WT plants grown in the field. Dry weight mean values are for all aboveground biomass, including the grain yield. (e) *pUbi:OsNRT2.1* transgenic lines and WT. (f) *pOsNAR2.1:OsNRT2.1* transgenic lines and WT. Statistical analysis was performed on data derived from the T3 generation. Error bars: SE (*n* = 3). Significant differences between the transgenic lines and WT are indicated by different letters (*P* < 0.05, one‐way ANOVA).

We analysed the grain yield and biomass of transgenic lines in the T0 and T1 generations. Relative to the wild‐type (WT) plants, the biomass, including the grain yield, of the 12 *pUbi:OsNRT2.1* lines increased by approximately 21.8% (Figure S2e) and 20.9% (Figure S3a) in T0 and T1 plants, respectively, but the grain yield decreased approximately 18.4% (Figure S2c) and 16.6% (Figure S3a) in T0 and T1 plants, respectively. Relative to the WT, the biomass, including the grain yield, of the 11 *pOsNAR2.1:OsNRT2.1* lines increased by average values of 32.2% (Figure S2f) and 27.1% (Figure S3b) in T0 and T1 plants, respectively, and the grain yield increased by average values of 30.7% (Figure S2d) and 28.1% (Figure S3b) in T0 and T1 plants, respectively. Based on the Southern blot analysis of T1 plants (Figure S4) and RNA expression data for the T0 generation (Figure S2a,b), we selected three independent *pUbi:OsNRT2.1* T1 lines OE1–2, OE2–5 and OE3–4 [renamed as OE1, OE2 and OE3 (Figure 1a)] and three independent *pOsNAR2.1:OsNRT2.1* T1 lines O6–4, O7–6 and O8–3 [renamed as O6, O7 and O8 (Figure 1b)].

Agricultural traits of these six lines were investigated in the field in the T1 through T4 generations, with a particular focus on the T3 generation. *OsNRT2.1* expression in roots was enhanced four‐ to sevenfold in the OE1, OE2 and OE3 lines but only 2.5‐ to threefold in the O6, O7 and O8 lines relative to the WT. In culms, *OsNRT2.1* expression was increased approximately sixfold in the OE lines and approximately threefold in the O lines. In leaf blades, however, only the OE lines exhibited increased *OsNRT2.1* expression (four‐ to sevenfold) compared with the WT, and no change in the expression was observed in the O lines (Figure 1c,d). The field data showed that both the OE and O lines exhibited increased growth and biomass, but only the O lines produced higher yields than the WT (Figure 1e,f).

Based on the agricultural traits of the T1–T4 generation plants in the field, the total aboveground biomass including the grain yield increased by 21% for the *pUbi:OsNRT2.1* lines and by 38% for the *pOsNAR2.1:OsNRT2.1* lines, while the biomass without grain yield increased by 190% for the *pUbi:OsNRT2.1* lines and by 160% for the *pOsNAR2.1:OsNRT2.1* lines. The grain yields of the *pUbi:OsNRT2.1* lines decreased over the three successive generations (Table 1), but the yields of the *pOsNAR2.1:OsNRT2.1* lines increased significantly from the T1 to T3 generation (Table 1). The yields of the O lines were enhanced by approximately 33% in T1 plants grown at Ledong and by 34%–42% in the T2 and T3 generations grown at Nanjing relative to the WT, while the OE lines exhibited lower yields than the WT by approximately 17% in all three generations (Table 1). We also analysed the yield and the biomass of the WT and T4 generation transgenic plants at Nanjing under low (180 kg N/ha) and normal N (300 kg N/ha) supplies. At the level of 180 kg N/ha, compared with WT, the yield of OE lines was reduced by 17%, and the biomass increased by 14%, while the yield and biomass of O lines were increased by 25% and 27% (Figure S5a), respectively. At the level of 300 kg N/ha, the yield of OE lines was reduced by 16%, and the biomass increased by 12%, as for O lines the yield and biomass were increased by 21% and 22%, respectively, compared with WT (Figure S5b).

**Table 1:** Comparison of the grain yield, dry weight and agronomic nitrogen‐use efficiency (ANUE) between the wild‐type (WT) and transgenic lines in the T1–T3 generations

	WT	*pUBi:OsNRT2.1*			*pOsNAR2.1:OsNRT2.1*		
		OE1	OE2	OE3	O6	O7	O8
Grain yield (kg/m^2^)							
T1	0.52 b	0.42 c	0.44 c	0.43 c	0.69 a	0.69 a	0.71 a
T2	0.66 b	0.54 c	0.56 c	0.54 c	0.89 a	0.91 a	0.93 a
T3	0.70 b	0.58 c	0.60 c	0.57 c	0.94 a	0.98 a	1.00 a
Dry weight (kg/m^2^)							
T1	1.05 c	1.31 b	1.29 b	1.31 b	1.43 a	1.45 a	1.47 a
T2	1.27 c	1.55 b	1.61 b	1.58 b	1.77 a	1.83 a	1.77 a
T3	1.40 c	1.67 b	1.73 b	1.69 b	1.91 a	1.96 a	1.95 a
ANUE (g/g)							
T1	15.48 b	12.43 c	12.94 c	12.56 c	19.64 a	19.63 a	19.86 a
T2	20.28 b	16.46 c	17.02 c	16.25 c	26.41 a	26.71 a	27.50 a
T3	21.33 b	17.42 c	18.41 c	17.01 c	26.17 a	27.86 a	28.12 a

Dry weight mean values are for all aboveground biomass, including the grain yield. For each mean, *n* = 3. Significant differences between the transgenic lines and WT are indicated by different letters (*P* < 0.05, one‐way ANOVA).

The total tiller number per plant in the T3 generation at the harvest stage increased 27.1% on average for both *pOsNAR2.1:OsNRT2.1* and *pUbi:OsNRT2.1* transgenic plants relative to the WT with no difference between the transgenic lines (Table 2); however, the grain number per panicle differed significantly between the OE and O lines (Table 2). The grain number per panicle increased approximately 15% in the O lines; the panicle length increased in the O lines approximately 12%, and the seed setting rate increased in the O lines by 14% relative to the WT (Table 2). The grain yields of the O lines increased by 24.2% relative to the WT (Table 2).

**Table 2:** Comparison of agronomic traits between the wild‐type (WT) and transgenic lines

Genotype	WT	*pUBi:OsNRT2.1*			*pOsNAR2.1:OsNRT2.1*		
		OE1	OE2	OE3	O6	O7	O8
Plant height (cm)	83.21 b	80.18 c	79.25 c	76.25 d	87.27 a	86.85 a	88.69 a
Total tiller number per plant	20.26 b	25.57 a	23.24 a	24.81 a	25.83 a	26.84 a	24.41 a
Panicle length (cm)	13.19 b	12.48 c	11.48 c	11.12 c	14.40 a	14.13 a	14.48 a
Grain weight (g/panicle)	2.22 d	1.16 e	0.96 f	1.23 e	3.87 a	2.74 c	3.01 b,c
Seed setting rate (%)	70.45 b	59.79 c	57.82 c	61.94 c	80.46 a	75.92 a	78.95 a
Grain number per panicle	132.58 b	105.67 c	97.25 c	101.61 c	154.50 a	166.25 a	149.75 a
1000‐grain weight (g)	25.24 a	24.89 a	24.39 a	24.45 a	25.28 a	25.67 a	25.89 a
Grain yield (g/plant)	26.21 b	21.61 c	22.63 c	21.19 c	31.17 a	32.81 a	33.64 a

Statistical analysis was performed on data derived from the T3 generation. Significant differences between the transgenic lines and WT are indicated by different letters (*P* < 0.05, one‐way ANOVA, *n* = 3).

### Nitrogen‐use efficiency of transgenic lines

Because the biomass and yields increased in the *pOsNAR2.1:OsNRT2.1* transgenic plants, we also analysed ANUE in T1–T4 generations of transgenic plants, N recovery efficiency (NRE), physiological N‐use efficiency (PNUE) and N harvest index (NHI) traits at the harvest stage in T3 generation transgenic lines to determine whether N use was altered in these plants, as modified the calculation method of the reference in Zhang *et al*. (2009). The ANUE of the O lines was enhanced by approximately 33% in T1 plants grown at Ledong and by 34%–42% in the T2 and T3 generations grown at Nanjing relative to the WT, while the OE lines exhibited lower ANUE than the WT by approximately 17% in all three generations (Table 1). In T4 plants at Nanjing, at the level of 180 kg N/ha, compared with WT, the ANUE of OE lines was reduced by 22% and the ANUE of O lines was increased by 33%, and at the level of 300 kg N/ha, the ANUE of OE lines was reduced by 17% and the ANUE of O lines was increased by 28% (Figure S5c). In the OE lines, the NRE increased to approximately 115% of the WT, and the PNUE and NHI were reduced to approximately 71% of the WT values. In the O lines, the ANUE increased to approximately 128% of the WT, the NRE increased to approximately 136% of the WT, and the PNUE and NHI were not significantly different from WT values (Table 4).

**Figure 2:**
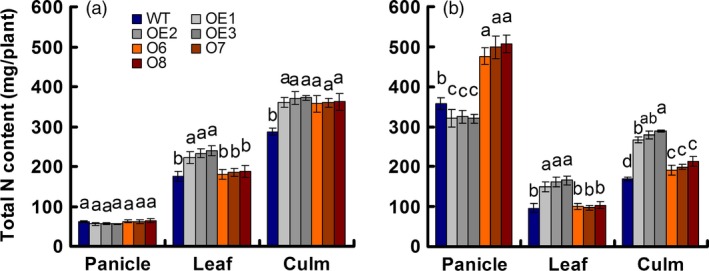
N content in various parts of the wild‐type (WT) and transgenic plants at two growth stages. (a) Sixty days after transplant, anthesis stage. (b) Ninety days after transplant, maturity stage. Error bars: SE (*n* = 3). Statistical analysis was performed on data derived from the T3 generation. Significant differences between the transgenic lines and WT are indicated by different letters (*P* < 0.05, one‐way ANOVA).

We sampled shoot tissues at the anthesis stage (60 days after transplanting) and the mature stage (90 days after transplanting) to determine the total N content. At the anthesis stage, total N was concentrated mainly in the culm with no difference between the OE and O lines, but with an increase of approximately 27% relative to the WT. In leaves, the total N content was the same in the O and WT lines, but was approximately 33% higher in the OE lines. The total N content in the grain was the same in all lines (Figure 2a). At the mature stage, total N was concentrated mainly in the grain, with the N content decreased by approximately 10% in the OE lines and increased by approximately 38% in the O lines relative to the WT (Figure 2b).

### Translocation of dry matter and N in transgenic lines

We investigated the dry matter and N translocation (NT) in rice plants by determining dry matter at anthesis (DMA), dry matter at maturity (DMM), total N accumulation at anthesis (TNAA) and total N accumulation at maturity (TNAM). For the OE lines, the DMA, the DMM, the TNAA and the TNAM increased by approximately 27%, 21%, 25% and 21%, respectively, relative to the WT. For the O lines, the DMA, the DMM, the TNAA and the TNAM increased by approximately 46%, 38%, 15% and 27%, respectively, relative to the WT (Table 3).

**Table 3:** Comparison of dry matter accumulation and N content between the wild‐type (WT) and transgenic lines

Dry matter and nitrogen components	WT	*pUBi:OsNRT2.1*			*pOsNAR2.1:OsNRT2.1*		
		OE1	OE2	OE3	O6	O7	O8
Dry matter at anthesis (kg/m^2^)	0.90 c	1.14 b	1.15 b	1.14 b	1.30 a	1.31 a	1.35 a
Dry matter at maturity (kg/m^2^)	1.40 c	1.67 b	1.78 b	1.69 b	1.91 a	1.96 a	1.95 a
Total nitrogen accumulation at anthesis (g/m^2^)	13.98 c	17.02 a	17.62 a	17.83 a	16.02 b	16.20 b	16.38 b
Total nitrogen accumulation at maturity (g/m^2^)	16.62 b	19.68 a	20.45 a	20.67 a	20.47 a	21.22 a	21.98 a
Grain nitrogen accumulation at maturity (g/m^2^)	9.56 b	8.56 c	8.67 c	8.54 c	12.69 a	13.30 a	13.53 a

Statistical analysis was performed on data derived from the T3 generation. For each mean, *n* = 3. Significant differences between the transgenic lines and WT are indicated by different letters (*P* < 0.05, one‐way ANOVA).

We also investigated the dry matter translocation (DMT), the DMT efficiency (DMTE), the contribution of preanthesis assimilates to grain yield (CPAY) and the harvest index (HI), based on the calculation method of the reference in Ntanos and Koutroubas (2002). For the OE lines, the DMT, DMTE, CPAY and HI decreased by approximately 68%, 75%, 61% and 31%, respectively, relative to the WT. For the O lines, the DMT increased by approximately 46%, while the DMTE, CPAY and HI did not differ between the O lines and the WT (Table 4).

**Table 4:** Comparison of N‐use efficiency, dry matter transport efficiency and N transport efficiency between the wild‐type (WT) and transgenic rice lines

	WT	*pUBi:OsNRT2.1*			*pOsNAR2.1:OsNRT2.1*		
		OE1	OE2	OE3	O6	O7	O8
N recovery efficiency (%)	39.06 c	44.59 b	45.40 b	45.64 b	52.13 a	53.29 a	53.68 a
Physiological N‐use efficiency (g/g)	54.55 a	39.96 b	40.56 b	37.26 b	50.10 a	51.49 a	52.40 a
N harvest index (%)	59.49 a	43.52 b	42.39 b	41.31 b	61.98 a	62.68 a	61.56 a
Dry matter transfer (g/m^2^)	198.95 c	72.25 d	51.03 e	67.74 d	301.22 a	278.87 b	293.48 a,b
Dry matter transfer efficiency (%)	22.10 a	6.32 b	4.45 c	5.95 b	23.23 a	21.22 a	21.78 a
Contribution of preanthesis assimilates to grain yield (%)	28.45 a	12.53 b	8.45 c	11.98 b	30.10 a	28.39 a	29.22 a
Harvest index (%)	49.93 a	34.46 b	34.96 b	33.47 b	49.20 a	50.34 a	51.46 a
Postanthesis N uptake (g/m^2^)	2.64 c	2.66 c	2.83 c	2.84 c	4.45 b	5.03 a	5.40 a
N translocation (g/m^2^)	6.91 b	5.91 c	5.84 c	5.70 c	8.24 a	8.28 a	7.93 a
N translocation efficiency (%)	49.45 a	34.69 b	33.14 b	31.97 b	51.42 a	51.10 a	48.42 a
Contribution of preanthesis N to grain N accumulation (%)	72.34 a	68.95 a	68.36 a	69.76 a	61.93 b	62.21 b	58.62 b

Statistical analysis was performed on data derived from the T3 generation. Methods of calculations in Table S4. For each mean, *n* = 3. Significant differences between the transgenic lines and WT are indicated by different letters (*P* < 0.05, one‐way ANOVA).

We investigated postanthesis N uptake (PANU), NT, N translocation efficiency (NTE) and the contribution of preanthesis N to grain N accumulation (CPNGN), as modified the calculation method of the reference in Ntanos and Koutroubas (2002) and Zhang *et al*. (2009). The PANU and CPNGN did not differ between the OE lines and the WT, but the NT and the NTE decreased by approximately 16% and 32%, respectively, in the OE lines relative to the WT. The NTE did not differ between the O lines and the WT, while the PANU and NT increased by approximately 87% and 18%, respectively, and the CPNGN decreased by approximately 16% in the O lines relative to the WT (Table 4).

### Expression patterns of *OsNRT2.1* and *OsNAR2.1* in different organs of the WT and transgenic lines

**Figure 3:**
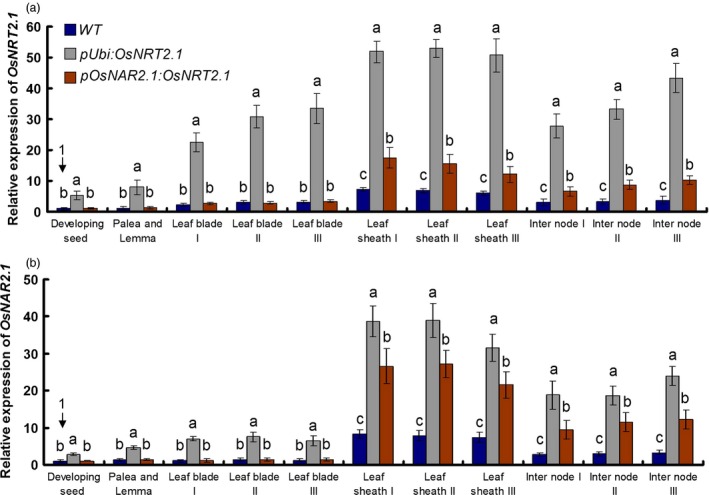
Expression pattern of *OsNRT2.1* and *OsNAR2.1*. Relative expression of (a) *OsNRT2.1* and (b) *OsNAR2.1* in various organs at 14 days after pollination. *pUbi:OsNRT2.1* represents the average of OE1, OE2 and OE3. *pOsNAR2.1:OsNRT2.1* represents the average of O6, O7 and O8. Statistical analysis was performed on data derived from the T4 generation. We defined that developing seed of the wild‐type (WT) expression was set equal to 1. Error bars: SE (*n* = 3). Significant differences between the transgenic lines and WT are indicated by different letters (*P* < 0.05, one‐way ANOVA).

Rice was previously shown to have a two‐component ${\text{NO}}_{3}^{-}$ uptake system consisting of *OsNRT2.1* and *OsNAR2.1*, similar to the system in *Arabidopsis* (Feng *et al*., 2011; Liu *et al*., 2014; Yan *et al*., 2011). We analysed the *OsNRT2.1* and *OsNAR2.1* expression patterns in the WT and transgenic lines during the filling stage. The detail about RNA samples was described in Figure S6 and Experimental procedures. The *OsNRT2.1* expression pattern in the WT showed that *OsNRT2.1* gene expressed most in root, secondly in leaf sheaths, thirdly in leaf blades and internodes and lest in grain including seed, palea and lemma (Table S5, Figure 3a). As for *OsNAR2.1,* it was expressed also most in root, secondly in leaf sheaths, thirdly in internodes and lest in grain and leaf blades (Table S5, Figure 3b). The coexpression pattern of *OsNRT2.1* and *OsNAR2.1* happened in root, leaf sheaths, internodes and grain but not in leaf blades (Table S5, Figure S7).

Compared with WT, the *OsNRT2.1* expression increased by about 7.5‐fold averagely in all organs of OE lines including root. The increase pattern of *OsNRT2.1* in OE lines showed the similar trade as the native expression of *OsNRT2.1* in the WT that was most in root, secondly in leaf sheaths, thirdly in leaf blades and internodes and lest in grain (Table S5, Figure 3a). It was very interesting that we found that in OE lines, the *OsNAR2.1* was also increased with the pattern as most in root, secondly in leaf sheaths, thirdly in internodes, fourthly in leaf blades and lest in grain (Table S5, Figure 3b). The coexpression increase pattern of *OsNRT2.1* and *OsNAR2.1* occurred in all organs of OE lines (Table S5, Figure S7).

Compared with WT, the *OsNRT2.1* expression was not changed in grain and leaf blades in O lines and increased in leaf sheaths, internodes and root significantly with a same pattern as WT, which is most in root, secondly in leaf sheaths, thirdly in internodes, fourthly in leaf blades and lest in grain (Table S5, Figure 3a). For *OsNAR2.1* expression in O lines, it was also not increased in grain and leaf blades only increased in leaf sheaths, internodes and root significantly with a same pattern as WT, which was most in root, secondly in leaf sheaths, thirdly in internodes and lest in grain and leaf blades (Table S5, Figure 3b). The coexpression increase pattern of *OsNRT2.1* and *OsNAR2.1* occurred in leaf sheaths, internodes and root of O lines (Table S5, Figure S7).

### Expression patterns of *OsNRT2.1* and *OsNAR2.1* in different growth stages of the WT and transgenic lines

**Figure 4:**
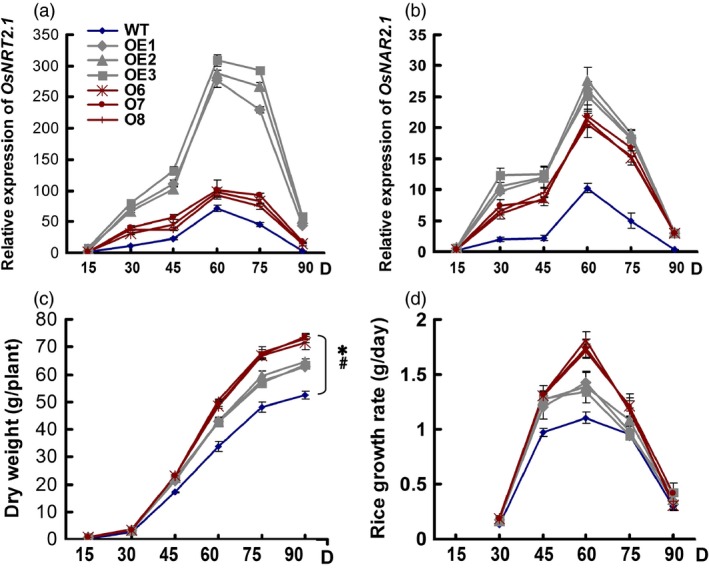
Growth status of the wild‐type (WT) and transgenic lines during the experimental growth period. (a) Changes in *OsNRT2.1* expression over the experimental growth period. (b) Changes in *OsNAR2.1* expression over the experimental growth period. RNA was extracted from culms. (c) Dry weight. Dry weight mean values are for all aboveground biomass, including the grain yield. (d) Growth rate. Samples were collected at 15‐day intervals after seedlings were transplanted to the field. Statistical analysis was performed on data derived from the T3 generation. Error bars: SE (*n* = 3). D in *x*‐axis means the day after transplanting. The asterisk at the end of time course indicates their statistically significant differences among plants, and #statistically significant differences during the growth stages (*P* < 0.05, ANCOVA).

In this study, we found that the *OsNRT2.1* and *OsNAR2.1* mRNA levels in the culms including the leaf sheath and internode (Figure S8) were significantly higher in all of the transgenic plants than in the WT plants (Figure 4a,b). *OsNRT2.1* expression was 3–20‐fold higher in the OE lines than in the WT, but was only 31%–45% higher in the O lines than in the WT (Figure 4a). *OsNAR2.1* expression was two‐ to ninefold higher in the OE lines than in the WT and was one‐ to eightfold higher in the O lines than in the WT (Figure 4b). Throughout the experimental growth period, *OsNRT2.1* expression was significantly higher in the culms of the OE lines than the O lines, but no significant difference in *OsNAR2.1* expression was observed between the OE and O transgenic lines.

During the entire experimental growth period, no significant differences in the *OsNRT2.1* and *OsNAR2.1* expression were found between the leaf blade I of the O lines and WT plants, but the expression levels of both *OsNRT2.1* and *OsNAR2.1* were up‐regulated significantly in the OE plants relative to the WT (Figure S9).

### Growth rate in transgenic lines

N transport and the growth of rice biomass are closely related, and *OsNRT2.1* overexpression was previously shown to affect the rice growth (Katayama *et al*., 2009). In this study, the OE and O lines began to show significantly higher biomass than WT plants at 45 days after transplanting and had accumulated 21% and 38% more biomass at 90 days (Figure 4c). The growth rates of the OE and O lines reached peak values at 60 days and were higher than those of the WT plants (Figure 4d). The growth rates of the OE and O lines were approximately 25% and 58% greater, respectively, than the WT. The growth rates of the transgenic and WT plants were identical after 75 days during the grain filling stage (Figure 4d).

### The coexpression of *OsNRT2.1* and *OsNAR2.1* in the WT and transgenic plants

The expression pattern of *OsNRT2.1* and *OsNAR2.1* in different organs showed that there exists a strong coexpression pattern of these two genes in rice plants (Figure S7). The coexpression pattern of *OsNRT2.1* and *OsNAR2.1* was altered very much in OE lines compared with O and WT lines (Figure S7). The expression ratio of *OsNRT2.1* and *OsNAR2.1* is 5.4 : 1 in the OE organs and 3.6 : 1 in the O lines compared with 3.9 : 1 in the WT organs (Figure S7). Furthermore, we specially investigated the ratio of *OsNAR2.1* to *OsNRT2.1* expression in root as 6.3 : 1 in the OE lines, 4.1 : 1 in the O lines and 4.2 : 1 in the WT plants, with no significant differences between the O lines and WT plants (Table S5).

**Figure 5:**
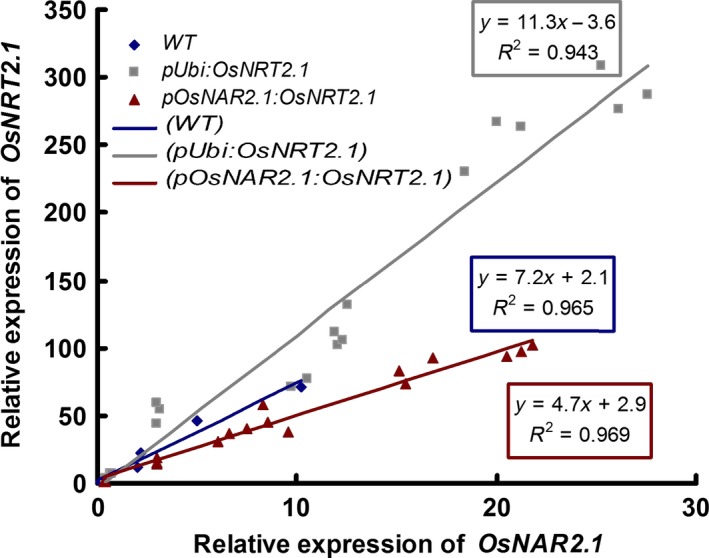
Ratios of *OsNRT2.1* to *OsNAR2.1* expression in culms of the wild‐type (WT) and transgenic lines over the course of the study. The ratios of *OsNRT2.1* and *OsNAR2.1* expression during different periods in the culms of *pUbi:OsNRT2.1* lines (OE1, OE2 and OE3), *pOsNAR2.1:OsNRT2.1* lines (O6, O7 and O8) and WT were presented.

The culm is important for N storage and translocation in rice shoots. In rice shoot, *OsNRT2.1* and *OsNAR2.1* expression was expressed most in leaf sheaths of culm (Figure 3). Our expression data also confirmed that *OsNRT2.1* and *OsNAR2.1* expression in the culm could play a key role in ${\text{NO}}_{3}^{-}$ remobilization. To further study the possible relationship between *OsNRT2.1* and *OsNAR2.1* expression and rice growth, we compared the ratio of *OsNRT2.1* and *OsNAR2.1* expression in rice plants. The expression ratio was approximately 11.3 : 1 in the OE lines and approximately 4.7 : 1 in the O lines compared with approximately 7.2 : 1 in the WT plants (Figure 5). We also investigated the ratio of *OsNAR2.1* to *OsNRT2.1* expression in leaf blade I. The expression ratio was 7.3 : 1 in the OE lines, 4 : 1 in the O lines and 5.2 : 1 in the WT plants, with no significant differences between the O lines and WT plants (Figure S10). The ratio of *OsNAR2.1* to *OsNRT2.1* expression correlated with the grain yield.

## Discussion

N nutrition affects all levels of plant function, from metabolism to resource allocation, growth and development (Crawford, 1995; Scheible *et al*., 1997, 2004; Stitt, 1999). As one form of available N nutrient to plants, ${\text{NO}}_{3}^{-}$ is taken up in the roots by active transport processes and stored in vacuoles in rice shoots (Fan *et al*., 2007; Li *et al*., 2008). In rice, OsNAR2.1 acts as a partner protein with OsNRT2.1 in the uptake and transport of ${\text{NO}}_{3}^{-}$ (Liu *et al*., 2014; Tang *et al*., 2012; Yan *et al*., 2011). *OsNAR2.1* gene expression was shown to be up‐regulated by ${\text{NO}}_{3}^{-}$ and down‐regulated by ${\text{NH}}_{4}^{+}$ (Feng *et al*., 2011; Nazoa *et al*., 2003; Zhuo *et al*., 1999).

Rooke *et al*. (2000) reported that the maize Ubi‐1 promoter had strong activity in young, metabolically active tissues and in pollen grains. Furthermore, Cornejo *et al*. (1993) performed histochemical localization of Ubi‐GUS activity and showed that the Ubi promoter was most active in rapidly dividing cells; however, Chen *et al*. (2012) reported that the Ubi promoter drove strong *OsPIN2* expression in all tissues. Chen *et al*. (2015) reported that ectopic expression of the *WOX11* gene driven by the promoter of the *OsHAK16* gene, which encodes a potassium (K) transporter that is induced by low K levels, led to an extensive root system, adventitious roots and increased tiller numbers in rice. In contrast, *WOX11* overexpression driven by the Ubi promoter induced ectopic crown roots in rice and failed to present any similar super growth phenotype in field (Zhao *et al*., 2009) as described by Chen *et al*. (2015). These results suggested that the use of a specific inducible promoter‐driven gene function could be a good strategy for plant breeding.

In this study, *OsNRT2.1* expression was up‐regulated significantly in both the aboveground and underground parts of *pUbi:OsNRT2.1* transgenic plants relative to the WT (Figure 1c), while *OsNRT2.1* expression in *pOsNAR2.1:OsNRT2.1* transgenic plants was increased significantly only in the roots and culms and not enhanced significantly in the leaves (Figure 1d). Specific induction of expression by the *OsNAR2.1* promoter in rice roots and culms based on GUS fusion data has been reported previously (Feng *et al*., 2011); therefore, we investigated the effects of tissue‐specific induction of *OsNRT2.1* expression in roots and culms on plant growth and nitrogen‐use efficiency (NUE).

### Effect of *pOsNAR2.1:OsNRT2.1* expression on NUE in transgenic rice

N redistribution during the reproductive stage was shown to vary significantly among cultivars and under various N management strategies (Souza *et al*., 1998). Mae and Ohira (1981) reported that a major proportion of N was redistributed from vegetative organs to panicles during grain filling, 64% of which was derived from leaf blades and 36% from culms. The NTE values of the WT, *pUbi:OsNRT2.1* and *pOsNAR2.1:OsNRT2.1* plants were averagely 49.5%, 33.4% and 50.3%, indicating that N transfer from the shoots into grain was significantly less in *pUbi:OsNRT2.1* transgenic plants than in the WT or *pOsNAR2.1:OsNRT2.1* plants (Table 4). This lower level of N transfer from vegetative organs to grain during grain filling in *pUbi:OsNRT2.1* plants affected spike formation and final grain yield compared with the WT and *pOsNAR2.1:OsNRT2.1* plants (Table 1). The DMTE values for WT, *pUbi:OsNRT2.1* and *pOsNAR2.1:OsNRT2.1* plants were 22.1%, 5.5% and 22.1%, averagely (Table 4) demonstrating that markedly less dry matter was transferred into grain yield in the *pUbi:OsNRT2.1* lines. These data confirmed that the transport of N and biomass during the transition from the flowering to harvest stages affected the final yield and NUE of rice (Zhang *et al*., 2009) and also indicated that the Ubi promoter decreased N and biomass translocation, while the *OsNAR2.1* promoter did not.

In both types of *OsNRT2.1* overexpression line, NT was reduced during the reproductive stage and NUE was reduced before flowering. The CPAY average values of the WT, *pUbi:OsNRT2.1* and *pOsNAR2.1:OsNRT2.1* plants were 28.5%, 11% and 34.9%, respectively. The CPAY of the *pOsNAR2.1:OsNRT2.1* plants was higher than that of the WT plants that had higher CPAY than the *pUbi:OsNRT2.1* plants (Table 4). The HI was much lower for the *pUbi:OsNRT2.1* plants than for the WT or *pOsNAR2.1:OsNRT2.1* plants (Table 4), indicating that the Ubi promoter affected ${\text{NO}}_{3}^{-}$ uptake and N use before the flowering stage and that levels of *OsNRT2.1* overexpression in rice that were excessive did not benefit N use during either the vegetative or reproductive stages.

### The coexpression pattern of *OsNRT2.1* and *OsNAR2.1* is an important factor controlling N transport in rice

How to assess the effect of ${\text{NO}}_{3}^{-}$ transporter expression on rice NUE is a key question for rice breeding. The ${\text{NO}}_{3}^{-}$ transporter, OsNRT1.1B, was shown to improve the NUE of rice by approximately 30% (Hu *et al*., 2015), while our data showed that not the higher expression level of ${\text{NO}}_{3}^{-}$ transporter was relative to the higher yield and NUE of rice (Tables 1 and 4, and Figure 4). After determining the expression levels of *OsNRT2.1* and its partner gene, *OsNAR2.1*, we calculated the coexpression ratio of these genes in rice plants.

The coexpression pattern of *OsNRT2.1* and *OsNAR2.1* happened in the WT and transgenic plants (Figures 3 and 4, Table S5). However, the coexpression pattern of *OsNRT2.1* and *OsNAR2.1* was changed in OE lines compared with O and WT lines (Figure S7), which suggested that *OsNRT2.1* driven by different promoters had a different coexpression patterns with *OsNAR2.1*. But it is still not clear that why increasing *OsNRT2.1* expression would induce *OsNAR2.1* expression and what mechanism exists behind the coexpression pattern of *OsNRT2.1* and *OsNAR2.1* in the gene regulation.

However, the ratio changes of *OsNRT2.1* to *OsNAR2.1* expression may be a clue for the explanation of the rice growth and nitrogen‐use difference in the WT and transgenic lines. The ratio changes of *OsNRT2.1* to *OsNAR2.1* expression in different organs were increased significantly in *pUbi:OsNRT2.1* lines compared with WT and *pOsNAR2.1:OsNRT2.1* lines (Figure S7). Also during the growth stages, the ratio of *OsNRT2.1* to *OsNAR2.1* expression in culm (including the internode and leaf sheath) was increased in *pUbi:OsNRT2.1* lines compared with WT and the *pOsNAR2.1:OsNRT2.1* lines (Figure 5). These data indicated that the interaction between *OsNRT2.1* and *OsNAR2.1* in *pUbi:OsNRT2.1* plants differed from WT and that in the *pOsNAR2.1:OsNRT2.1* lines. Furthermore in culms, *pOsNAR2.1:OsNRT2.1* lines showed a lower expression ratio of these two genes, in which more OsNAR2.1 protein may be available to interact with OsNRT2.1 protein. Therefore, the efficiency of OsNRT2.1 function in rice plants should differ between the two types of transgenic plants resulting in different rice yield and NUE phenotypes. On the other hand, the higher expression of *OsNRT2.1* and *OsNAR2.1* in all the organs of pUbi:*OsNRT2.1* than WT may cause some disadvantages to plants such as high cost for mRNA synthesis or disturbing of nitrogen transport in the leaf blades. All possibilities remain to be confirmed by further analysis.

In this study, we showed that the rice yield and NUE could be improved by increasing *OsNRT2.1* expression, especially in combination with a lower expression ratio with its partner gene *OsNAR2.1*, which encodes a high‐affinity ${\text{NO}}_{3}^{-}$ transporter.

## Experimental procedures

### Construction of vectors and rice transformation

We amplified the *OsNRT2.1/OsNRT2.2* ORF sequence, which is identical for both genes, from cDNA isolated from *Oryza sativa* L. ssp. Japonica cv. Nipponbare using the primers listed in Table S1. We amplified the *OsNAR2.1* and ubiquitin promoters from the *pOsNAR2.1(1698bp):GUS* (Feng *et al*., 2011) and *pUbi:OsPIN2* (Chen *et al*., 2012) constructs, respectively, using the primers listed in Table S2. The PCR products were cloned into the pMD19‐T vector (TaKaRa Biotechnology, Dalian, China) and confirmed by restriction enzyme digestion and DNA sequencing. The *pUbi:OsNRT2.1* and *pOsNAR2.1:OsNRT2.1* vectors were constructed as shown in Figure S1. These constructs were introduced into *Agrobacterium tumefaciens* strain EHA105 by electroporation and then transformed into rice as described previously (Tang *et al*., 2012).

### Southern blot analysis

Transgene copy number was determined by the Southern blot analysis following procedures described previously (Jia *et al*., 2011). Briefly, genomic DNA was extracted from the leaves of wild type (WT) and digested with *Hin*dIII and *Eco*RI restriction enzymes. The digested DNA was separated on a 1% (w/v) agarose gel, transferred to a Hybond‐N^+^ nylon membrane and hybridized with hygromycin‐resistant gene.

### Biomass, total nitrogen (N) measurement and calculation of NUE

WT and transgenic rice plants were harvested at 9:00 a.m. and heated at 105 °C for 30 min. Panicles, leaves and culms were then dried at 75 °C for 3 days. Dry weights were recorded as biomass values. Samples collected at 15‐day intervals from WT and transgenic lines grown in soil in pots were used to calculate whole‐plant biomass values.

Total N content was measured using the Kjeldahl method (Li *et al*., 2006). The total dry weight (biomass) was estimated as the sum of weights of all plant parts. Total N accumulation was estimated as the sum of the N contents of all plant parts. Agronomic NUE (ANUE, g/g) was calculated as (grain yield − grain yield of zero N plot)/N supply; NRE (%) was calculated as (total N accumulation at maturity for N‐treated plot − total N accumulation at maturity of zero N plot)/N supply; physiological NUE (PNUE, g/g) was calculated as (grain yield − grain yield of zero N plot)/total N accumulation at maturity; and the NHI (%) was calculated as (grain N accumulation at maturity/total N accumulation at maturity). Dry matter and NT and translocation efficiency method for the calculation of the reference in Ntanos and Koutroubas (2002) and Zhang *et al*. (2009). Dry matter translocation (DMT, g/m^2^) was calculated as dry matter at anthesis − (dry matter at maturity − grain yield); dry matter translocation efficiency (DMTE, %) was calculated as (dry matter translocation/dry matter at anthesis) × 100%; the CPAY (%) was calculated as (dry matter translocation/grain yield) × 100%; the HI (%) was calculated as (grain yield/dry matter at maturity) × 100%; PANU (g/m^2^) was calculated as total N accumulation at maturity − total N accumulation at anthesis; NT (g/m^2^) was calculated as total N accumulation at anthesis − (total N accumulation at maturity − grain N accumulation at maturity); NTE (%) was calculated as (N translocation/total N accumulation at anthesis) × 100%; and the CPNGN (%) was calculated as (N translocation/grain N accumulation at maturity) × 100% (Table S4).

### The growth conditions for T0 to T4 transgenic plants

T0, T2, T3 and T4 generation plants were grown in plots at the Nanjing Agricultural University in Nanjing, Jiangsu (Figure S11). T1 generation plants were grown in Sanya, Hainan. Jiangsu is in a subtropical monsoon climate zone. Chemical properties of the soils in the plots at the Nanjing Agricultural University included organic matter, 11.56 g/kg; total N content, 0.91 g/kg; available P content, 18.91 mg/kg; exchangeable K, 185.67 mg/kg; and pH 6.5. Basal applications of 30 kg P/ha (Ca(H_2_PO_4_)_2_) and 60 kg/K ha (KCl) were made to all plots 3 days before transplanting. N fertilizer accounted for 40%, 30% and 40% of the total N fertilizer was applied prior to transplanting, at tillering, just before the heading stage, respectively.

### The field experiments for yield harvest

T0–T4 generation seedlings were planted in the same experiment site in Nanjing, except T1 in Sanya. Seed generation transgenic lines and WT were surface‐sterilized with 10% (v : v) hydrogen peroxide (H_2_O_2_) for 30 min and rinsed thoroughly with deionized water. The transgenic seeds were soaked in water containing 25 mg/L hygromycin, and the WT seeds were soaked in water. After 3 days, the sterilized seeds were sown evenly in wet soil. The similar seedlings were transplanted to field plots after 3 weeks of germination.

T1–T3 plants were planted in plots fertilized at a rate of 300 kg N/ha as urea and in plots without N fertilization. Plots were 2 × 2.5 m in size with the seedlings planted in a 10 × 10 array. Plants at the edges of all four sides of each plot were removed at maturity to avoid the influence of edge effects. Four points, each containing four seedlings, totally 16 seedlings, were selected randomly within the remaining centre 8 × 8 array of plants, and samples were collected (Khuram *et al*., 2013; Ookawa *et al*., 2010; Pan *et al*., 2013; Srikanth *et al*., 2016). Yield and biomass values determined from these four points in each plot were used to calculate the yield per hectare and biomass of each line, and three random plots for each line were designed in the experiment (Figure S11).

T3 generation plants were sampled at 15‐day intervals for the determination of the grain yield, biomass and N content. The growth rate was the dry weight of the weight increase in the unit time after seedlings were transplanted to the plots.

T4 generation plants were planted in a plot fertilized at a rate of 0, 180 and 300 kg N/ha as urea. Same random field plots with three replicates were designed as T1–T3 plants for yield, and biomass values determined from these four points were used to calculate the yield and biomass per plant and ANUE of each line.

### mRNA sampling and qRT‐PCR assay

To investigate the expression pattern in plant organs, we sampled mRNA for seeds, palea and lemma, leaf blade I, leaf blade II, leaf blade III, leaf sheath I, leaf sheath II, leaf sheath III, internode I, internode II, internode III and newly developed root (3 cm from root tips) at the grain filling stage (described in Figure S6). Tracking rice in the whole growth period of gene expression in T3 generation, we sampled mRNA from culms including leaf sheath and internode I (described in Figure S8) at 15, 30, 45, 60, 75 and 90 days after transplanting.

Total RNAs were prepared from the various tissues of the WT and transgenic plants using TRIzol reagent (Vazyme Biotech Co., Ltd, http://www.vazyme.com). Real‐time PCR was carried as described before (Li *et al*., 2014). All primers used for qRT‐PCR are listed in Table S3.

### Statistical analysis

Data were analysed by Tukey's test of one‐way analysis of variance (ANOVA), except that analysis of covariate (ANCOVA) was used in the biomass and growth rate during growth stages (Figure 4a,b). Different letters on the histograms or after mean values indicate statistically significant differences at *P* < 0.05 between the transgenic plants and WT (one‐way ANOVA). The asterisk at the end of time course indicates their statistically significant differences among plants, and #statistically significant differences during the growth stages at *P* < 0.05 (ANCOVA). All statistical evaluations were conducted using the IBM SPSS Statistics version 20 software (SPSS Inc., Chicago, IL).

## Supporting information


**Figure S1** Diagram of (a) *pUbi:OsNRT2.1* and (b) *pOsNAR2.1:OsNRT2.1* constructs.
**Figure S2** Characterization of T0 generation transgenic lines.
**Figure S3** Grain yield and dry weight of WT and T1 generation transgenic plants.
**Figure S4** Southern blot analysis of transgene copy number.
**Figure S5** Grain yield, dry weight and ANUE of WT and T4 generation transgenic plants under low and normal N supplies.
**Figure S6** The diagram of RNA sampling in T4 generation transgenic lines and WT plants.
**Figure S7** Ratios of *OsNRT2.1* to *OsNAR2.1* expression in different organs of WT and transgenic lines.
**Figure S8** The diagram of RNA sampling in T3 generation transgenic lines and WT plants.
**Figure S9** Changes in genes expression in leaf blade I throughout the experimental growth period.
**Figure S10** Ratios of *OsNRT2.1* and *OsNAR2.1* expression in the leaf blade I of WT and transgenic plants during different periods.
**Figure S11** A field experiment picture of WT and T3 generation transgenic plants.
**Table S1** Primers used to amplify the *OsNRT2.1* open reading frame.
**Table S2** Primers used to amplify the *OsNAR2.1* and *Ubiquitin* promoters.
**Table S3** Primers used to detect *OsActin*,* OsNAR2.1*, and *OsNRT2.1* gene expression.
**Table S4** Methods of NUE calculations.
**Table S5** Real‐time quantitative RT‐PCR analysis of endogenous *OsNRT2.1* and *OsNAR2.1* expression in various transgenic lines and wild‐type (WT) plants.
