# Supplementary material for: Agronomic nitrogen‐use efficiency of rice can be increased by driving OsNRT2.1 expression with the OsNAR2.1 promoter
Source: Plant Biotechnol J. 2016 Jan 30;14(8):1705–15. doi: 10.1111/pbi.12531 (PMC5066696; doi:10.1111/pbi.12531)

Table S1. Primers used to amplify the *OsNRT2.1* open reading frame.

Primer name	Forward primer (5'to3')	Reverse primer (5'to3')
OsNRT2.1	CCAAGGTACCATGGACTCGTCGACGGTGGGC	GGCCACTAGTTTAGGCGTGCTCCGGCGAG

Table S2. Primers used to amplify the *OsNAR2.1* and *Ubiquiton* promoters.

Primer name	Forward primer (5'to3')	Reverse primer (5'to3')
OsNAR2.1 Promoter	GAGGCGCGCCTGCTGACAAACCAAACCGACT	CATTAATTAACCCCACCTCTCCCACCTCAC
Ubiquiton Promoter	GGCGCGCCGTCGACCTGCAGGTCGAC	TTAATTAACTGCAGTGCAGCGTGACCC

Table S3. Primers used to detect *OsActin*, *OsNAR2.1*, and *OsNRT2.1* gene expression.

Gene name	Accession No.	Forward primer (5'to3')	Reverse primer (5'to3')
OsActin	AB047313	GGAACTGGTATGGTCAAGGC	AGTCTCATGGATAACCGCAG
OsNAR2.1	AP004023	GTCGTCGAGAAGCGCAAGA	GTCCACTGAAGCTGCGAACTT
OsNRT2.1	AB008519	AGCACCATGTTCAACAGCAAGA	CGAAGTAGGCCAGCCTCCAC

Table S4. Methods of NUE calculations

Components:	Expressed as:	Definition:
Agronomic nitrogen use efficiency	ANUE	Gw /Ns
Nitrogen recovery efficiency	NRE	RTNA /Ns ×100%
Physiological nitrogen use efficiency	PNUE	Gw /TNAM
Nitrogen harvest index	NHI	GNAM /TNAM ×100%
Dry matter translocation	DMT	DMA-(DMM-Grain Yield)
Dry matter translocation efficiency	DMTE	DMT/DMA ×100%
Contribution of pre-anthesis assimilates to grain yield	CPAY	DMT/Grain Yield ×100%
Harvest index	HI	Grain Yield/DMM ×100%
Post-anthesis N uptake	PANU	TNAM-TNAA
Nitrogen translocation	NT	TNAA-(TNAM-GNAM)
Nitrogen translocation efficiency	NTE	NT/TNAA ×100%
Contribution of pre-anthesis N to grain nitrogen accumulation	CPNGN	NT/GNAM ×100%

Note：Gw = grain yield – grain yield of zero-N plot; RTNA = TNAM of N-treated plot – TNAM of zero-N plot; Ns = N supply; TNAM = total N accumulation at maturity; GNAM = grain N accumulation at maturity.

Table S5. Real-time quantitative RT-PCR analysis of endogenous *OsNRT2.1* and *OsNAR2.1* expression in various transgenic lines and wild-type (WT) plants.

OsNRT2.1		*pUBi:OsNRT2.1*				*pOsNAR2.1:OsNRT2.1*			
Organs in plant	WT	OE1	OE2	OE3	I-fold	O6	O7	O8	I-fold
Seed	1.0	4.4	4.9	6.4	4.2	1.1	1.0	1.0	0
PL	1.2	5.6	7.7	10.3	5.5	1.4	1.4	1.3	0.1
Leaf blade I	2.2	22.8	18.2	26.5	9.0	2.7	2.9	2.5	0.2
Leaf blade II	2.9	29.2	30.6	32.7	9.4	2.7	2.7	3.3	0
Leaf blade III	3.2	27.9	31.9	40.8	9.5	3.4	3.5	3.5	0.1
Leaf sheath I	7.2	42.8	49.3	63.5	6.2	17.1	17.5	17.8	1.4
Leaf sheath II	6.8	45.7	49.9	63.1	6.7	15.6	15.7	15.1	1.3
Leaf sheath III	6.0	43.9	47.3	61.0	7.5	12.2	12.8	11.4	1
Inter node I	3.1	23.2	28	32.2	8	6.7	6.6	6.4	1.1
Inter node II	3.3	29.2	33.8	36.7	9.2	8.4	9.0	8.7	1.7
Inter node III	3.7	35.9	45.2	46.2	10.6	8.8	10.8	11.0	1.8
Root	21.1	98.7	110.7	151.2	4.7	52.5	55.8	58.7	1.6
OsNAR2.1		*pUBi:OsNRT2.1*				*pOsNAR2.1:OsNRT2.1*			
Organs in plant	WT	OE1	OE2	OE3	I-fold	O6	O7	O8	I-fold
Seed	0.3	0.6	0.7	0.8	1.8	0.3	0.2	0.2	0
PL	0.3	0.9	1.2	1.4	2.6	0.4	0.4	0.3	0.1
Leaf blade I	0.3	1.8	1.6	1.8	5.3	0.3	0.3	0.3	0.1
Leaf blade II	0.3	1.6	2	2.1	4.6	0.3	0.4	0.5	0.2
Leaf blade III	0.3	1.3	1.5	2.2	4.6	0.3	0.4	0.3	0.1
Leaf sheath I	2.1	9.3	9.5	10.5	3.6	6.7	6.7	6.8	2.2
Leaf sheath II	2.0	9.6	9.8	10.1	3.9	7.0	7.0	6.7	2.4
Leaf sheath III	1.8	7.4	7.8	8.7	3.3	5.5	5.5	5.3	1.9
Inter node I	0.7	4.3	4.66	5.3	5.8	2.4	2.4	2.4	2.4
Inter node II	0.7	4.4	4.3	5.4	5.4	2.9	2.9	2.9	2.9
Inter node III	0.8	5.3	6.2	6.7	6.2	3.0	3.0	3.2	2.7
Root	4.9	15.5	17.2	23.1	2.8	12.7	13.1	14.2	1.7

Note：I-fold: The average increase ford compared with WT. PL means palea and lemma. RNA was extracted from 14 days after pollination. We defined the *OsNRT21* expression of WT was set equal to 1

Figure S1. Diagram of *pUbi:OsNRT2.1* and *pOsNAR2.1:OsNRT2.1* constructs.

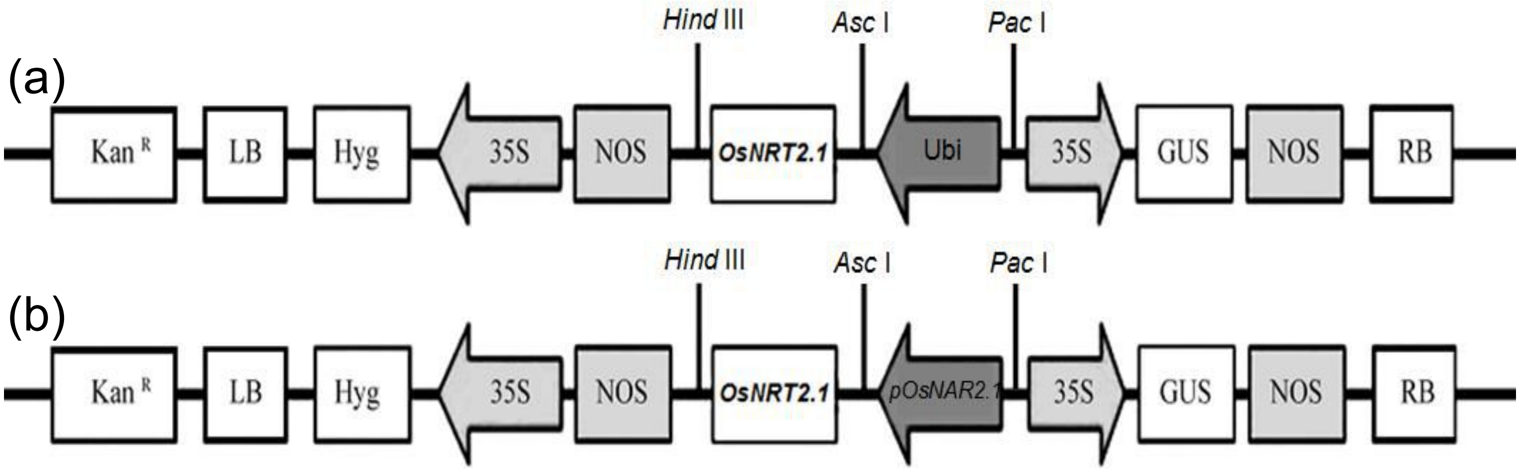

Figure S2. Characterization of T0 generation transgenic lines.

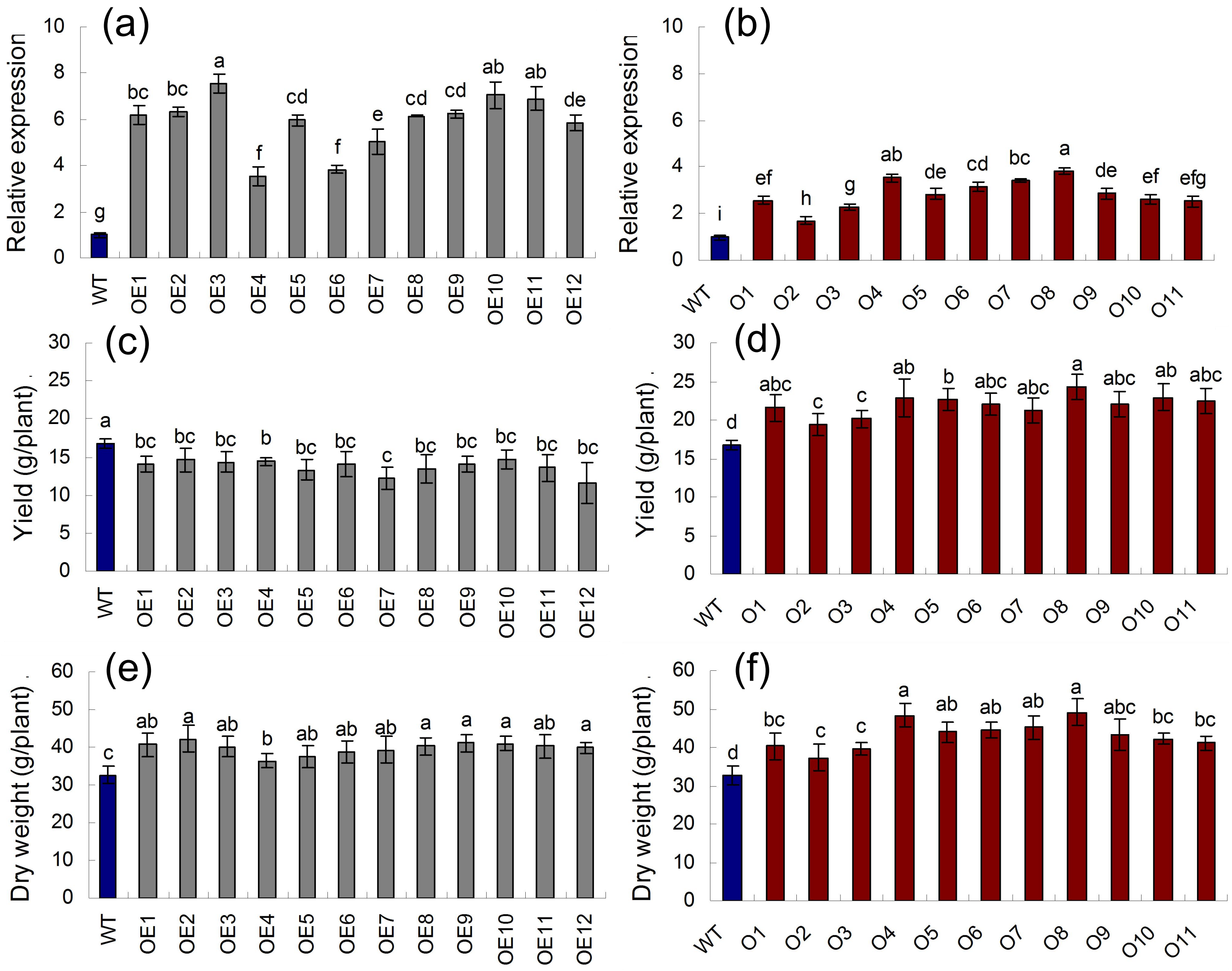

Figure S3. Grain yield and dry weight of WT and T1 generation transgenic plants.

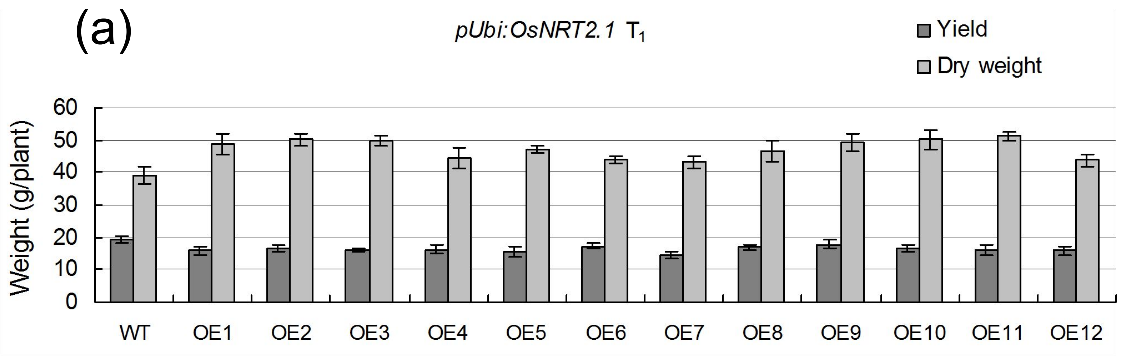

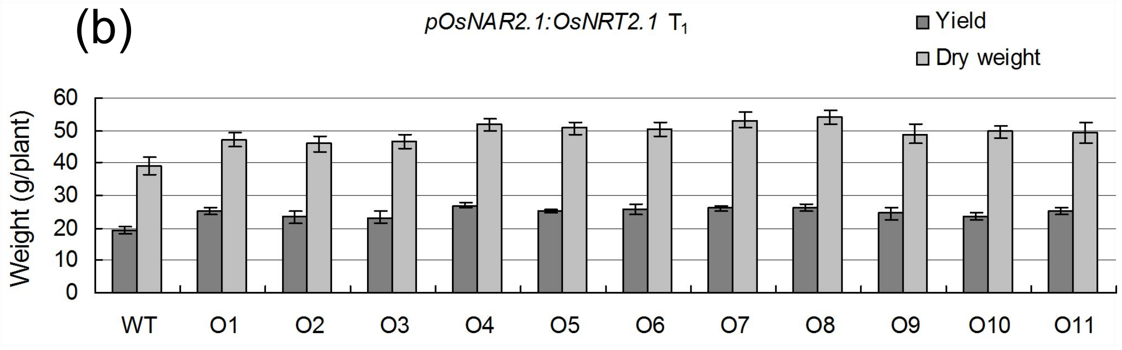

Figure S4. Southern blot analysis of transgene copy number.

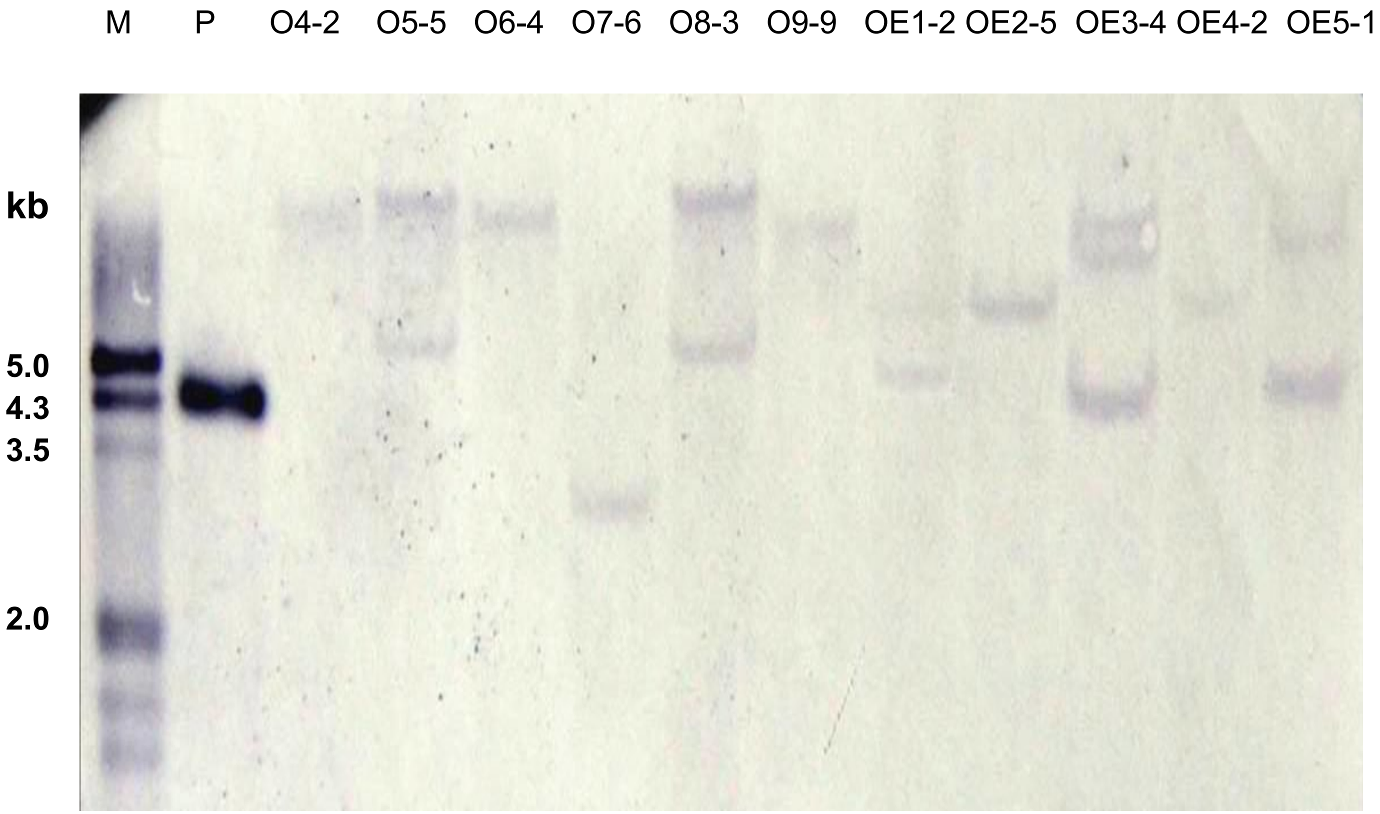

Figure S5. Grain yield, dry weight and ANUE of WT and T4 generation transgenic plants under low and normal N supplies .

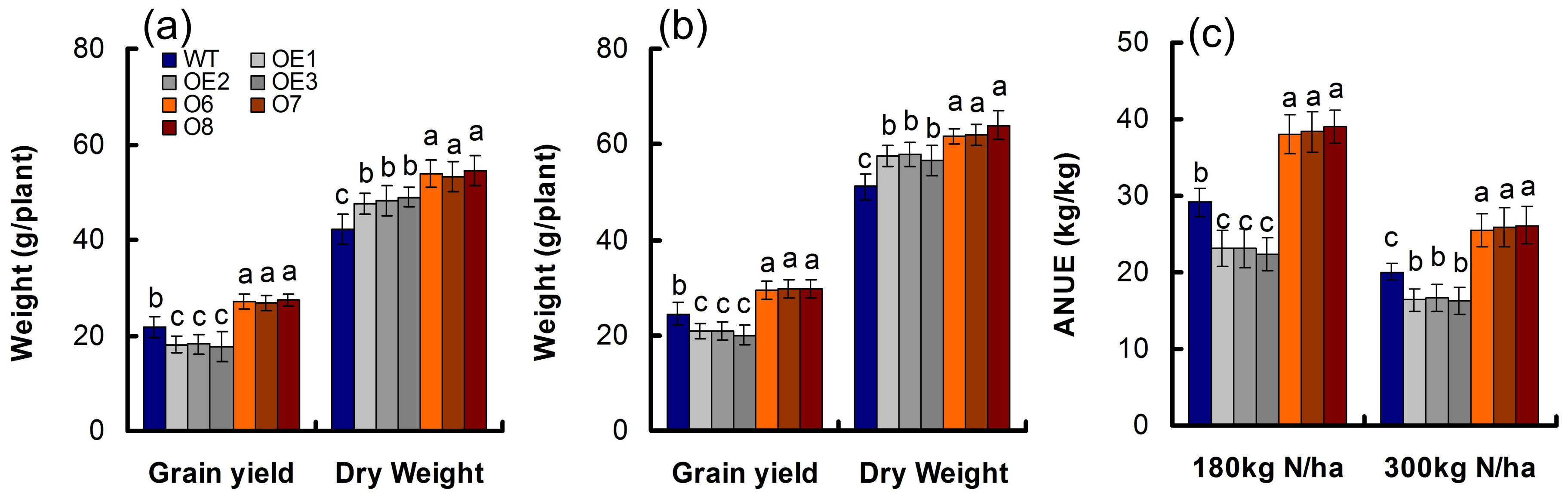

Figure S6. The diagramma of RNA sampling in T4 generation transgenic lines and WT plants.

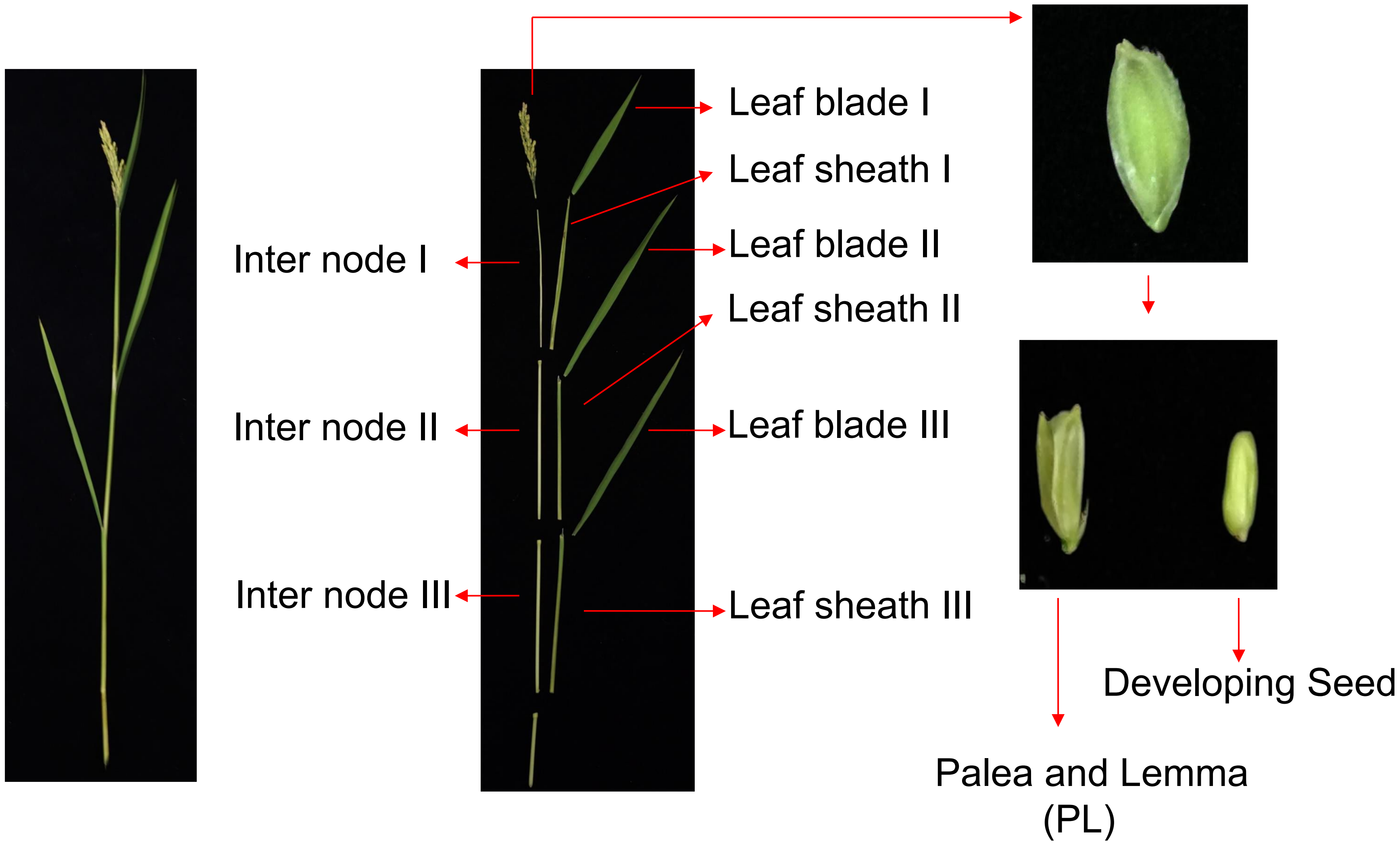

Figure S7. Ratios of *OsNRT2.1* to *OsNAR2.1* expression in different organs of WT and transgenic lines**.**

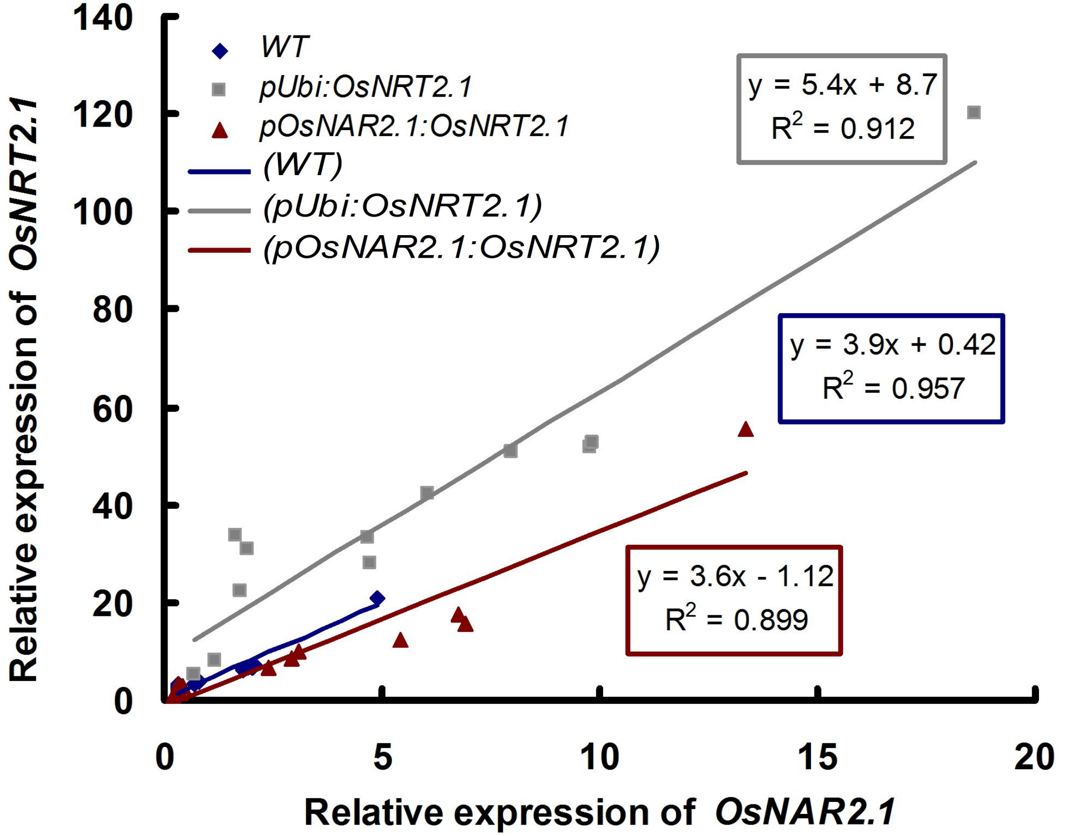

Figure S8. The diagramma of RNA sampling in T3 generation transgenic lines and WT plants.

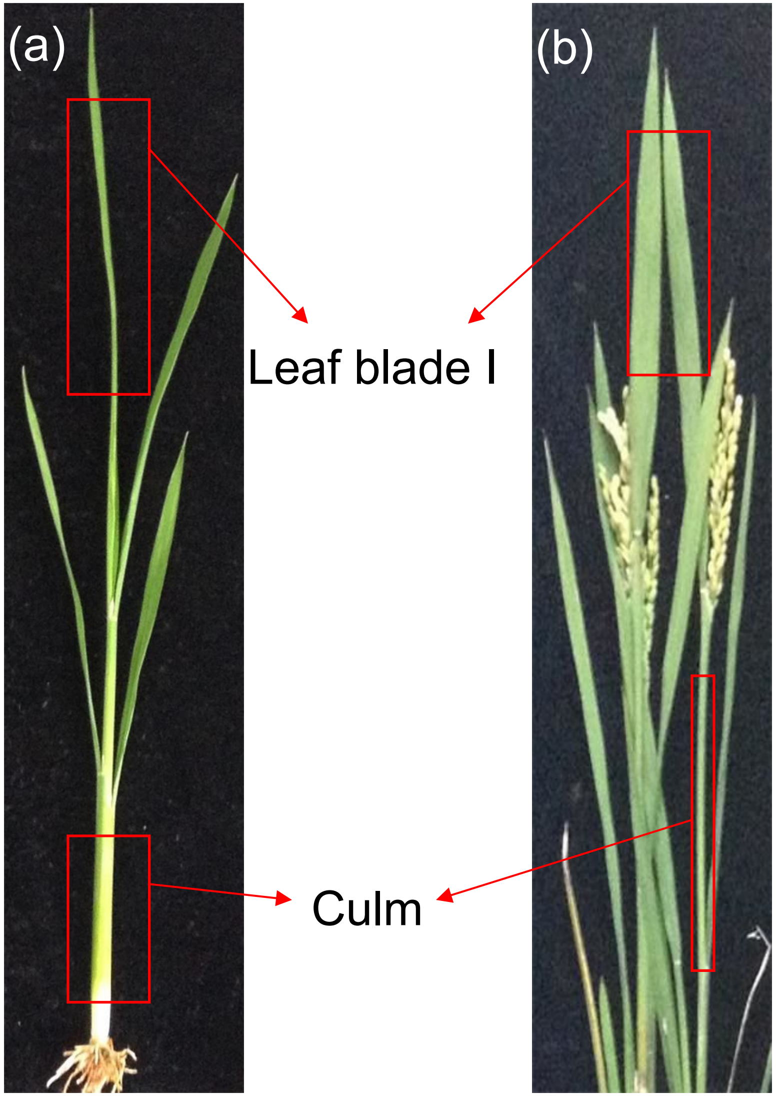

Figure S9. Changes in genes expression in leaf blade I throughout the experimental growth period.

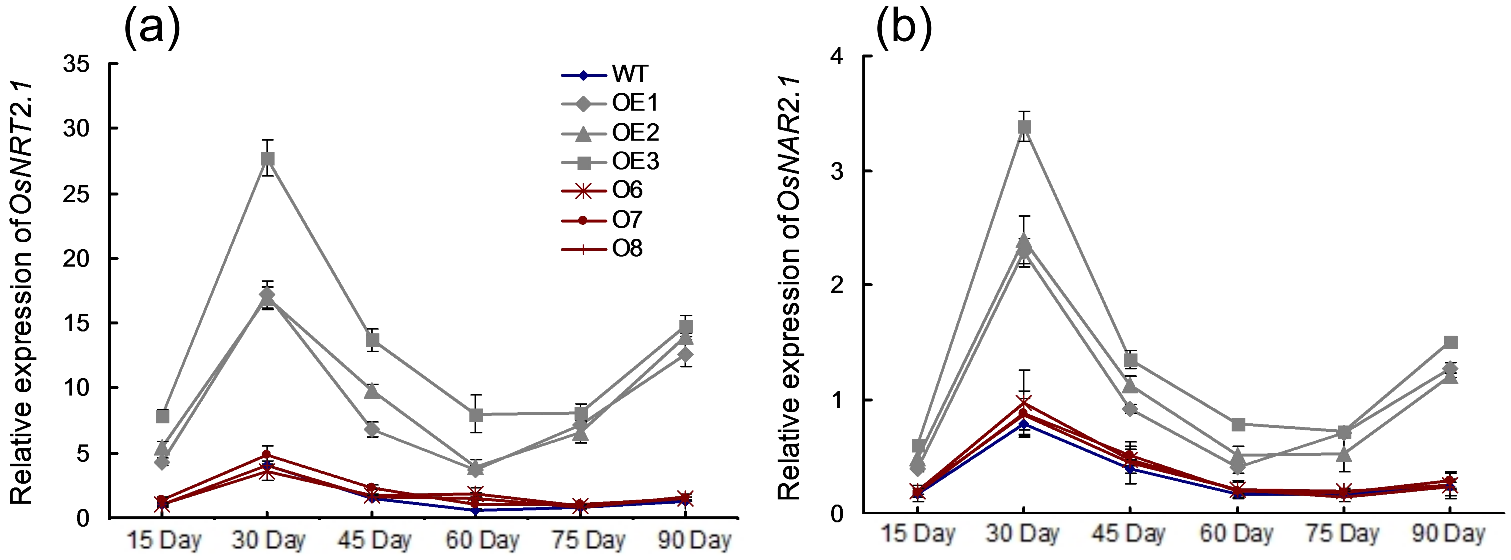

Figure S10. Ratios of *OsNRT2.1* and *OsNAR2.1* expression in the leaf blade I of WT and transgenic plants during different periods.

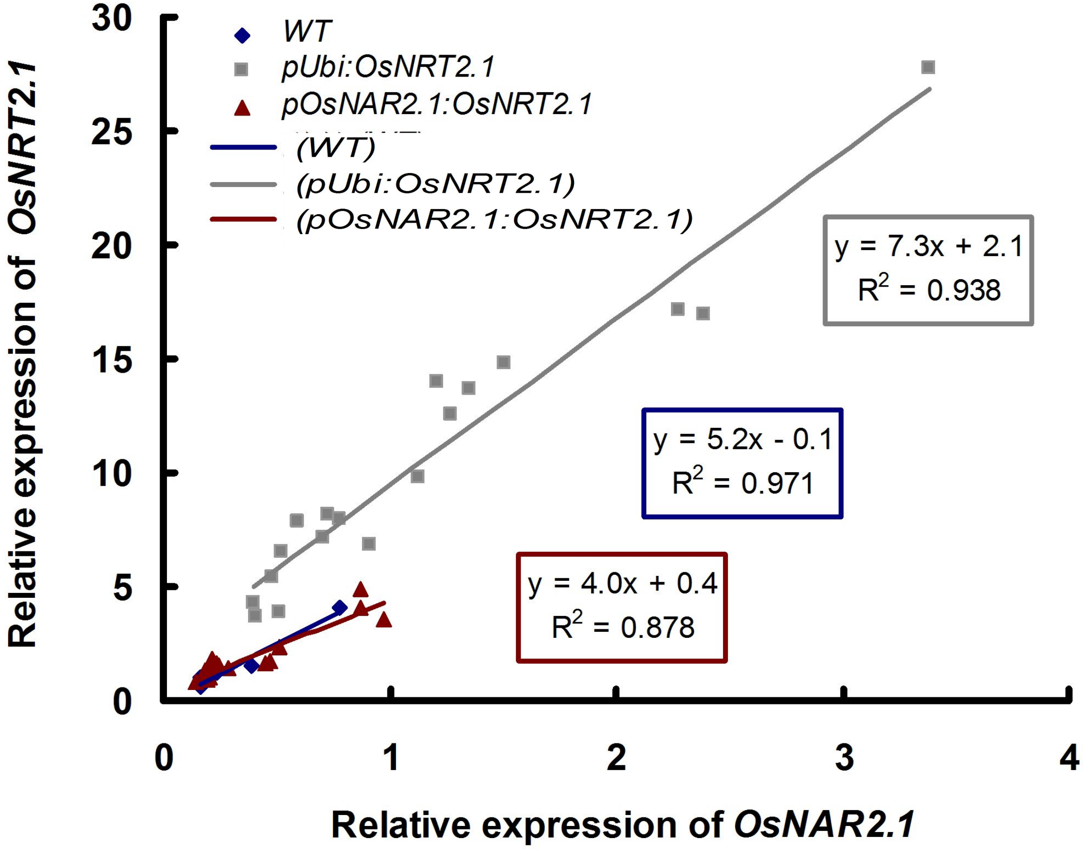

Figure S11. A field experiment picture of WT and T3 generation transgenic plants.

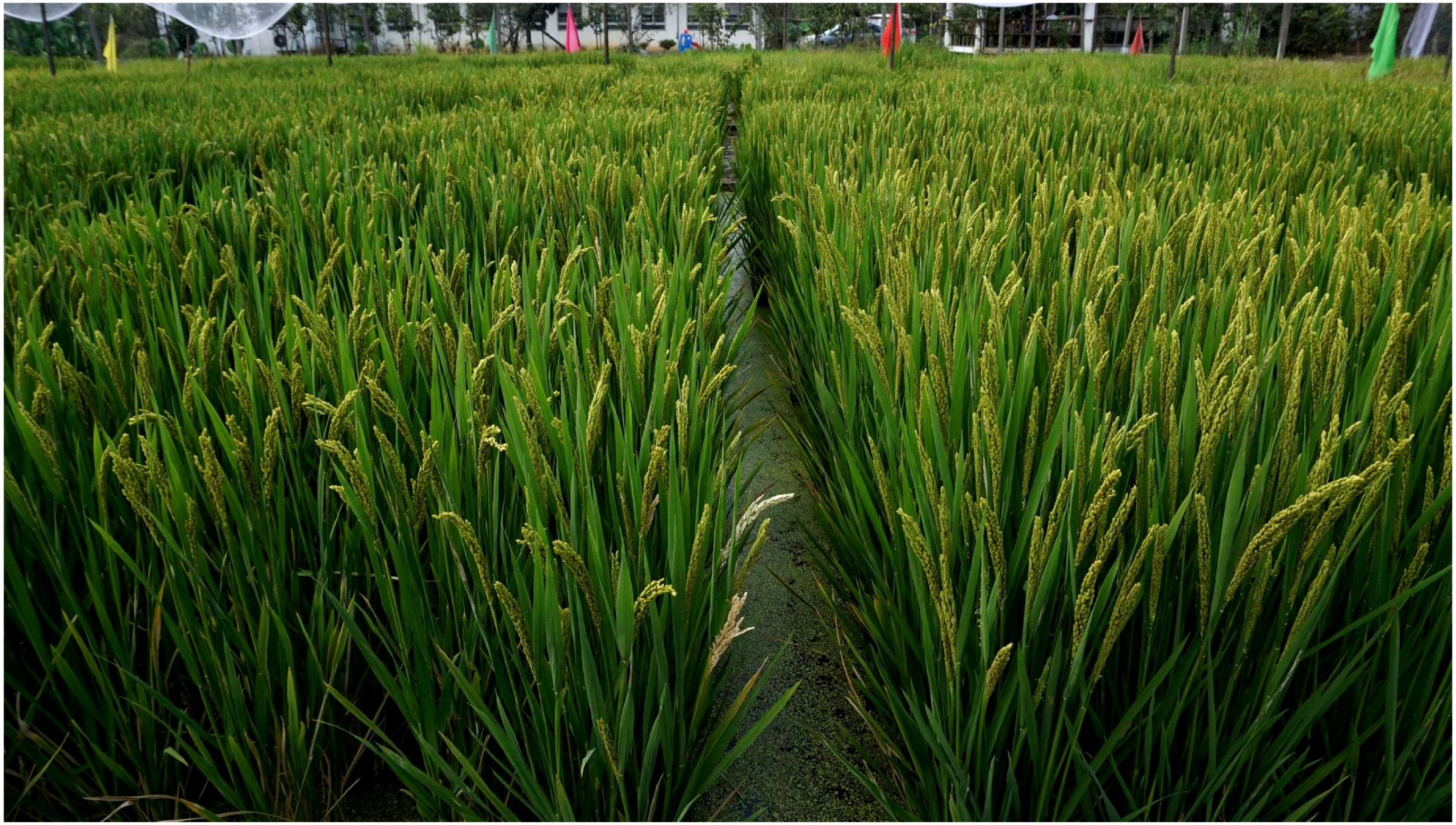

Supplement: Supplementary file 1 — Figure S1 Diagram of (a) pUbi:OsNRT2.1 and (b) pOsNAR2.1:OsNRT2.1 constructs. Figure S2 Characterization of T0 generation transgenic lines. Figure S3 Grain yield and dry weight of WT and T1 generation transgenic plants. Figure S4 Southern blot analysis of transgene copy number. Figure S5 Grain yield, dry weight and ANUE of WT and T4 generation transgenic plants under low and normal N supplies. Figure S6 The diagram of RNA sampling in T4 generation transgenic lines and WT plants. Figure S7 Ratios of OsNRT2.1 to OsNAR2.1 expression in different organs of WT and transgenic lines. Figure S8 The diagram of RNA sampling in T3 generation transgenic lines and WT plants. Figure S9 Changes in genes expression in leaf blade I throughout the experimental growth period. Figure S10 Ratios of OsNRT2.1 and OsNAR2.1 expression in the leaf blade I of WT and transgenic plants during different periods. Figure S11 A field experiment picture of WT and T3 generation transgenic plants. Table S1 Primers used to amplify the OsNRT2.1 open reading frame. Table S2 Primers used to amplify the OsNAR2.1 and Ubiquitin promoters. Table S3 Primers used to detect OsActin, OsNAR2.1, and OsNRT2.1 gene expression. Table S4 Methods of NUE calculations. Table S5 Real‐time quantitative RT‐PCR analysis of endogenous OsNRT2.1 and OsNAR2.1 expression in various transgenic lines and wild‐type (WT) plants. [file PBI-14-1705-s001.pdf]
